# Supplementary material for: Inhibitors of Rho/MRTF/SRF Transcription Pathway Regulate Mitochondrial Function
Source: Cells. 2024 Feb 24;13(5):392. doi: 10.3390/cells13050392 (PMC10931493; doi:10.3390/cells13050392)
Supplement: Supplementary file 1 [file cells-13-00392-s001.zip › cells-2827192-supplementary.pdf]

Supplementary Figures

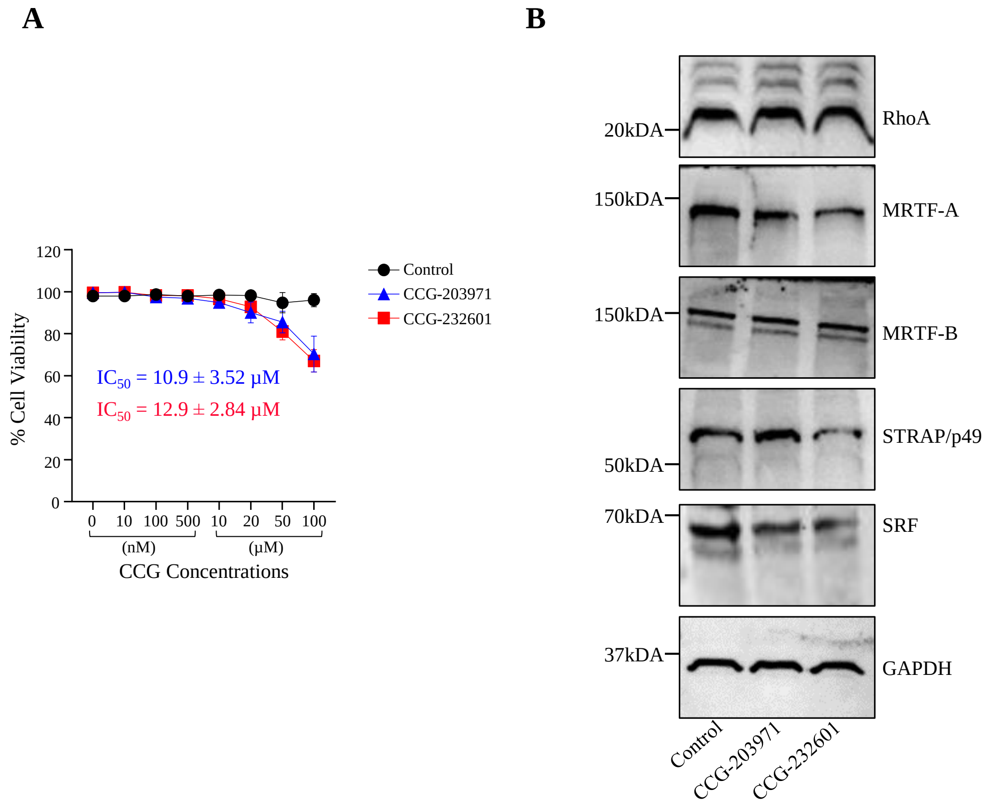

Figure S1. Cellular viability and inhibition of Rho/MRTF/SRF signaling in mouse myoblast cells. (**A**) MTS assay was performed on C2C12 cells treated with control and varying concentrations of CCG-203971 and CCG-232601 for 24h. Graphed values are shown as the mean ± SD ($n = 4$) and the calculated IC50 values are presented. (**B**) C2C12 cells were treated with 20 μM of CCG molecules for 24 h. Representative western blots show the protein expression of RhoA, MRTF-A, MRTF-B, STRAP/p49, and SRF; and GAPDH was used as a loading control.

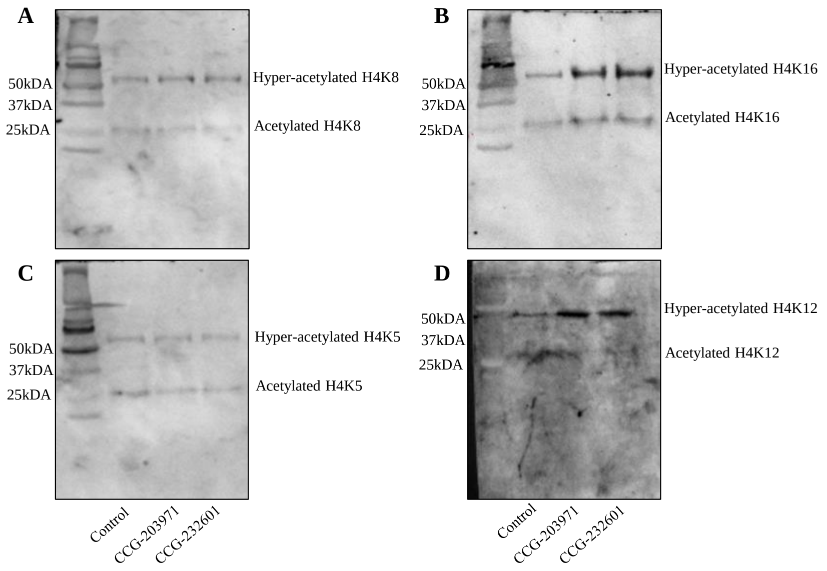

Figure S2. Immunoblots from Figure 3 demonstrate hyper-acetylated and acetylated H4 Lysines. WI-38 cells were treated with 20 μM of CCG molecules for 24 h. Western blot analysis was used to examine the expression levels of histone H4 acetylation. Hyper-acetylated H4 lysines were observed at high molecular weight ~ 50 kDA and acetylated H4 lysines were observed at low molecular weight ~ 25 kDA. Western blot image for (**A**) H4K8ac (**B**) H4K16ac (**C**) H4K5ac (**D**) H4K12ac. No change in the expression levels of hyper-acetylated and acetylated H4K8 and H4K5 was observed, whereas H4K12 and H4K16 induced hyper-acetylation.

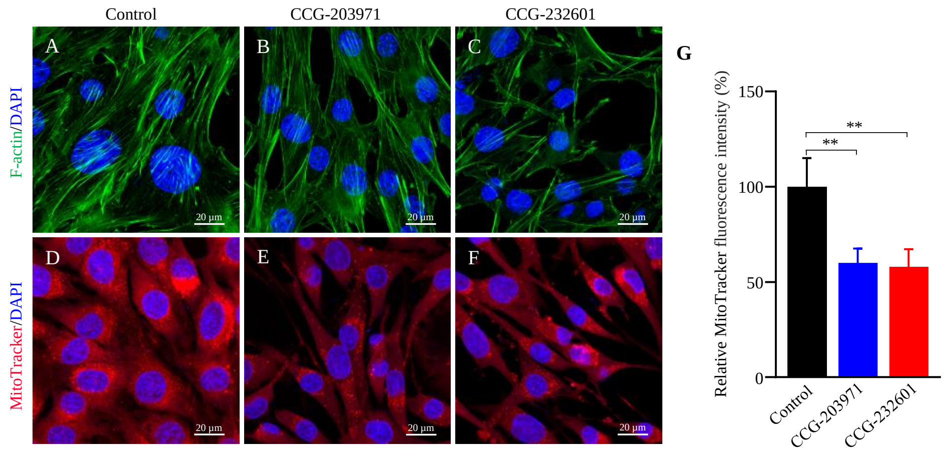

Figure S3. Immunolabeling of F-actin and MitoTracker Red CMXRos in C2C12 cells. C2C12 cells treated with 20 μM CCG molecules for 24 h and stained with phalloidin (green) and MitoTracker Red CMXRos conjugated dyes. DAPI (blue) was used for nuclear counterstaining. (**A**, **D**) Control (0.5% DMSO treated) (**B**, **E**) CCG-203971 treated (**C**, **F**) CCG-232601 treated. A 63× oil objective is used; scale bars indicate 20 μm. Images are representative of 3 repeats. (**G**) Bar graphs show a significant reduction in MitoTracker Red fluorescence intensity (normalized) in treated cells to control. Data plotted as mean ± SD. Data were pooled from 3 independent experiments (one-way ANOVA, ***p* < 0.01).

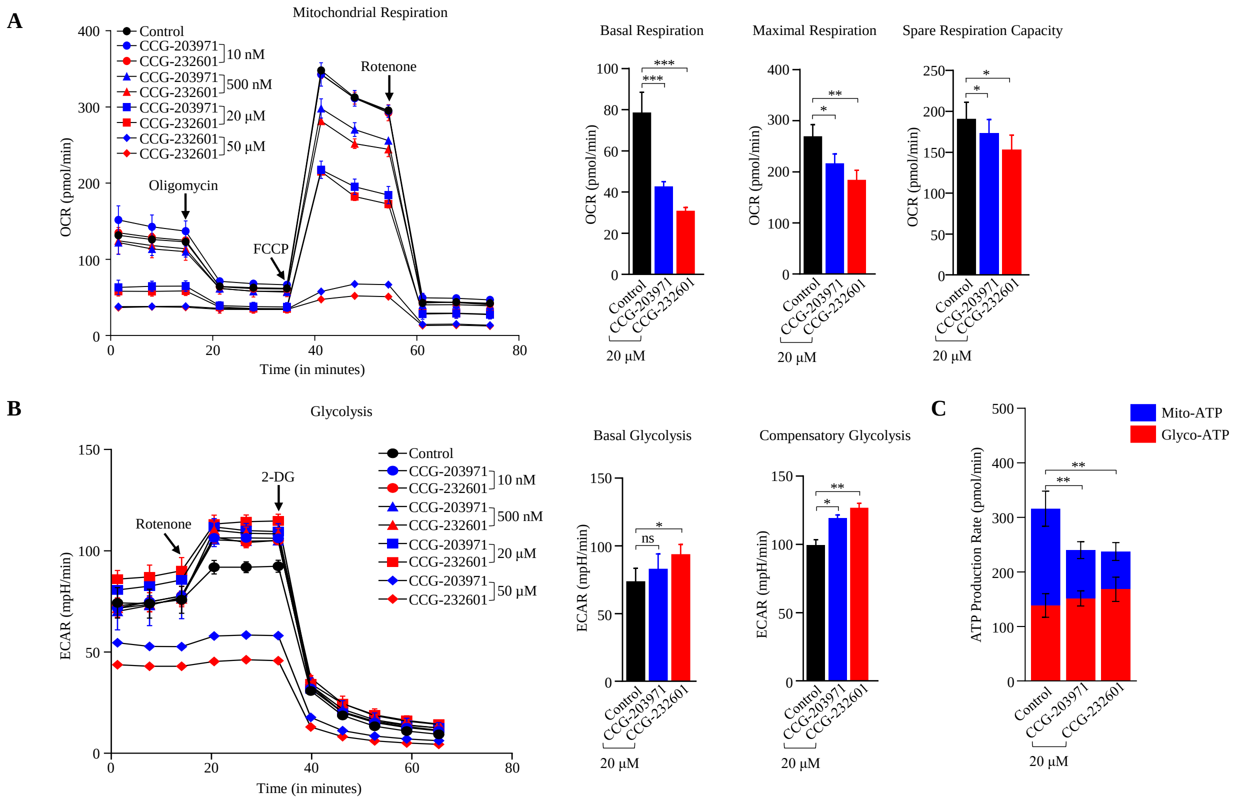

Figure S4. Effect of CCG molecules on OCR and ECAR profiles in C2C12 cells. C2C12 cells were treated with CCG-203971 and CCG-232601 at varying concentrations for 24 h (**A**) Seahorse XF Cell Mito Stress Test analysis of control (0.5% DMSO treated) and CCG-treated cells showing mean ± SD normalized to equal number of cells. Graphs show the quantification of basal respiration, maximal respiration, and spare respiratory capacity (**B**) Glycolytic rate assay shown as mean ± SD normalized to equal number of cells. Bar graphs show the calculated glycolytic parameters of basal and compensatory glycolysis. (**C**) ATP rate assay. There is reduction in total ATP production on treatment with CCG molecules, glyco-ATP increases while Mito-ATP decreases on the treatment. Error bars represent mean ± SD (n = 3). *p < 0.05; **$p < 0.01$; ***$p < 0.001$, ns, $p > 0.05$ by one-way ANOVA with Tukey's method.
